# Judgment Bias Responses in Domestic Dog Puppies at 60 Days of Age

**DOI:** 10.3390/vetsci13080828

**Published:** 2026-08-19

**Authors:** Valentina Gazzano, Virginia Bellini, Francesca Cecchi, Maria Claudia Curadi, Paolo Baragli, Angelo Gazzano, Asahi Ogi

**Affiliations:** 1 Department of Veterinary Sciences, University of Pisa, 56124 Pisa, Italy; valentina.gazzano@unipi.it (V.G.); virgy.bellini95@gmail.com (V.B.); francesca.cecchi@unipi.it (F.C.); paolo.baragli@unipi.it (P.B.); angelo.gazzano@unipi.it (A.G.); 2 Department of Clinical Sciences and Translational Medicine, University of Rome Tor Vergata, 00133 Rome, Italy; asahi.ogi@uniroma2.eu

**Keywords:** judgment bias, cognitive bias, puppy, *Canis familiaris*, affective state, animal welfare

## Abstract

Judgment bias tasks are widely used to assess affective state in adult dogs but have rarely been applied before adoption age. To test whether a spatial version is viable, 82 puppies from 17 litters were tested at 60 days of age; 80 provided valid observations for analysis. After discrimination training, positive (P), negative (N) and ambiguous (A) locations were presented in up to three trials; latency to the bowl was recorded (30 s cut-off). Latencies from the first two trials (476 observations, 85 right-censored) were analyzed with mixed-effects Cox proportional hazards models including puppy and litter as random intercepts. Median recorded times were 2.54 s (P), 13.25 s (N) and 3.86 s (A), with timeouts in 0%, 38.4% and 15.2%, respectively. Cue location affected approach rate (χ^2^ = 207.03, df = 2, *p* < 0.001); hazard ratios were N versus P 0.11 (95% CI 0.09–0.15), A versus P 0.32 (0.25–0.41) and N versus A 0.36 (0.27–0.47), all significant (Holm-adjusted). Trial number (*p* = 0.076) and sex (*p* = 0.483) were not significant. Random-effect variances were 0.180 (puppy) and 0.261 (litter). Puppies discriminated between outcome-associated locations and approached the ambiguous location at an intermediate rate, indicating that the task is feasible at 60 days of age.

## Simple Summary

How an animal feels is a central part of its welfare, but feelings cannot be measured directly. One way around this problem is to watch how an animal behaves when a situation is unclear: individuals in a more positive state tend to act as though something good is likely to happen, while those in a more negative state act as though it is not. This approach has been used with adult dogs, but almost nothing is known about very young puppies. We enrolled eighty-two puppies from seventeen litters at sixty days of age; eighty puppies provided valid test data for analysis. They were still living with their mother and littermates at the breeder and before moving to their new homes. Each puppy first learned that a bowl placed in one position contained food, and that a bowl placed in another position did not. It was then offered a bowl in a position halfway between the two. The puppies went quickly to the position where food was expected, much more slowly to the position where it was not, and at an intermediate speed to the uncertain position. No clear differences were detected between male and female puppies. These findings support the feasibility of using this type of test with very young puppies.

## 1. Introduction

An assessment of animals’ affective experiences is a central component of contemporary animal welfare science, since good welfare is now understood to require the presence of positive experiences rather than merely the absence of negative ones [1]. Traditional welfare indicators, including physiological measures and the observation of overt behavior, provide important information, but spontaneous responses of this kind largely reflect the intensity of an emotional experience rather than its valence, and may therefore fail to distinguish whether an animal is in a positive or a negative state [2,3]. Learned responses, by contrast, appear more informative about valence, and cognitive measures have accordingly been used with increasing frequency to investigate how affective state influences the processing and interpretation of environmental information [4]. One of the most widely applied approaches is the judgment bias task, which is based on the principle that affective state can influence expectations about uncertain outcomes [4,5].

In a typical judgment bias task, animals first learn that one stimulus or location predicts a positive outcome, usually access to a food reward, whereas another predicts the absence of reward or a less favorable outcome [6]. They are then presented with one or more intermediate, ambiguous stimuli, and their responses to these are compared with those to the trained cues [5]. A relatively rapid response to an ambiguous cue is generally interpreted as reflecting a stronger expectation of a positive outcome, whereas a slower response or failure to respond suggests a weaker expectation of reward. These response patterns are conventionally described as relatively “optimistic” or “pessimistic”, and this terminology is now widely established across taxa [7]. However, these terms refer to the animal’s response within a specific experimental context and should not automatically be considered stable personality characteristics [6]. More generally, the interpretation of these tasks is not unequivocal, and the conceptual assumptions underlying the inference from behavioral response to affective state remain debated [8].

The paradigm was first developed in rats, where animals housed in unpredictable conditions responded to ambiguous cues as though anticipating a less favorable outcome [9], and has since been extended to a wide range of taxa and progressively refined, for instance through automated and self-initiated procedures based on spontaneous investigative behavior [10]. It has proved sensitive both to acute affective manipulations, such as release from restraint in sheep [11], and to longer-term husbandry conditions, including environmental enrichment in pigs [12] and compromised welfare in horses [13]; pharmacological depletion of serotonin has likewise been shown to shift sheep towards more pessimistic responses [14]. Comparable responses have been documented even in fish [15]. In domestic dogs, judgment bias paradigms have been adapted for domestic dogs using spatial, visual, and auditory stimuli. In spatial tasks, dogs are required to discriminate between a rewarded and an unrewarded bowl location before their latency to approach bowls placed in intermediate positions is measured [16], whereas visual versions employ cues such as cards of intermediate shade [17] and auditory versions rely on tones of intermediate frequency, in some cases delivered by automated apparatus [18].

The first canine studies showed that dogs displaying separation-related behaviors approached ambiguous locations more slowly than dogs showing fewer signs of these problems, suggesting an association between judgment bias and underlying affective state [16]. Subsequent work has applied the paradigm to the effects of owner absence [19], of environmental and occupational enrichment [20], and of clinical intervention, with dogs treated for separation-related problems shifting towards less pessimistic responses over the course of treatment [21]. Judgment bias tasks have also been used to compare individual dogs and dog populations, including pet and shelter dogs [22,23]. Alongside these applications, several authors have proposed methodological revisions intended to make the task easier to learn and its outcomes more reliable, either by modifying the spatial paradigm itself [24] or by replacing arbitrary cues with ecologically relevant ones, thereby shortening the training phase [25].

Despite their potential, canine judgment bias tasks present several methodological challenges. Approach latency may be influenced not only by affective state but also by food motivation, locomotor activity, exploratory tendency, personality and previous learning [4,6], and dogs’ performance has been shown to depend on their learning history independently of welfare-related determinants [26]. In addition, ambiguous locations are generally not rewarded. Repeated presentations may therefore cause dogs to learn that these locations do not contain food, progressively reducing their ambiguity and increasing approach latency, a limitation of repeated judgment bias testing already documented in other species [27] and identified as a priority for methodological refinement [6]. Previous canine studies have reported individual differences in task performance and changes in responses following repeated exposure, highlighting the need to consider both individual variation and trial repetition when interpreting results [18,22,23]. A study specifically examining repeated testing in dogs found low reliability across sessions, progressively longer latencies and an increasing number of non-approaches, providing direct evidence that dogs learned that the ambiguous locations were unrewarded [28].

These constraints are particularly relevant when the paradigm is applied to very young subjects, for whom lengthy training and repeated exposure are least practicable. Most canine judgment bias research has in fact involved dogs older than the puppies examined in the present study. Nevertheless, several cognitive and social abilities required to participate in behavioral tests are already evident at approximately eight weeks of age. Puppies between eight and ten weeks succeed in visual, auditory and olfactory discrimination problems, retain the location of hidden food over short delays, and display emerging executive-function abilities, albeit at a lower level than adult dogs [29]. Responsiveness to human communicative gestures has also been reported at this age [29,30], although the age at which this ability becomes reliable remains debated [31,32]. Eight-week-old puppies from a multi-breed sample have also been shown to learn socially from both humans and conspecifics and to retain the acquired information over short intervals [33], although breed-related differences were not specifically investigated. Sixty days of age is also of particular methodological interest because it immediately precedes adoption: at this stage puppies are still living with their dam and littermates at the breeder’s premises and have not yet been exposed to the divergent home environments, management routines and training experiences that accompany placement in a new household. Testing at this age therefore reduces a source of between-individual variability that is difficult to control in studies of adult dogs.

To our knowledge, judgment bias responses have not previously been characterized in domestic dog puppies at 60 days of age. Because several cognitive traits measured in early puppyhood show moderate rank-order stability into young adulthood [34], characterizing responses at this stage may also provide a basis for future longitudinal studies examining whether early performance is associated with later behavioral characteristics or welfare outcomes.

The present study therefore investigated the responses of 60-day-old domestic dog puppies to positive, ambiguous and negative locations in a spatial judgment bias task. Approach latency and the probability of reaching each location within the established observation period were evaluated. We hypothesized that puppies would approach the positive location most rapidly, the negative location most slowly, and the ambiguous location at an intermediate rate. We also examined whether responses varied between repeated trials or according to sex, while accounting for repeated observations from the same puppy and for the shared background of puppies belonging to the same litter. Because no experimental manipulation of affective state was performed, the study was intended to characterize puppies’ responses to ambiguity at the group level rather than to classify individual puppies as stably optimistic or pessimistic.

## 2. Materials and Methods

### 2.1. Animals and Ethical Statement

Eighty-two puppies from 17 litters, representing 10 breeds, were recruited from seven breeding facilities, some of which contributed more than one litter. All puppies were tested at 60 days of age and were living with their dam and littermates at the breeder’s premises at the time of testing. Puppies were included only if they were clinically healthy, had already been weaned onto solid food, and had no previous experience with any behavioral test.

To ensure that performance was not limited by unfamiliarity with the test elements, breeders were instructed in advance to ensure that each puppy (i) was accustomed to eating from a stainless-steel bowl similar to those used in the test, (ii) had already experienced the type of food reward used in the test (small pieces of frankfurter sausages), and (iii) was familiar with the area in which the test would take place. Compliance with these requirements was confirmed with the breeder before testing.

Two puppies met the training criterion but did not maintain sufficient task engagement during the subsequent test phase; consequently, no valid test observations could be obtained for these subjects. They were therefore excluded from the behavioral analyses, leaving 80 puppies from 17 litters in the analytical sample (Table 1).

The order in which the puppies of each litter were tested was randomly determined by drawing lots. All puppies belonging to the same litter were tested consecutively on the same day, between 08:00 and 11:00, with each litter tested at approximately the same time of day. Data collection was conducted from February to May 2025 and 2026. Ambient temperature was not systematically recorded, but testing was conducted only under environmental conditions considered appropriate for the welfare and comfort of the animals. The puppies did not receive their morning meal before habituation and testing.

The study protocol was reviewed by the Animal Welfare Committee (OPBA) of the University of Pisa, which issued a favorable opinion on 12 February 2025 (resolution no. 14/2025). The procedures were classified as non-experimental veterinary clinical practices pursuant to Article 2, paragraph 1, letter (b), of Italian Legislative Decree 26/2014, implementing Directive 2010/63/EU. Written informed consent was obtained from all breeders/owners before participation.

### 2.2. Experimental Setting and Apparatus

Each litter was tested at its own breeding facility in an area already familiar to the puppies, selected to be quiet and free from major distractions. Testing was conducted either indoors or outdoors, depending on the facilities available at each breeding premises. The flooring was concrete, and the testing pen was enclosed by opaque plastic barriers. Although the overall dimensions of the available space differed slightly among facilities, a minimum area of 5 × 4 m was required, and the geometry relevant to the task was standardized across all sites. The release point and target locations were positioned according to fixed measurements and verified with a tape measure before each session. In addition, the sides assigned to the positive and negative locations were counterbalanced across puppies, reducing the possibility that a fixed spatial cue associated with one side of the arena systematically corresponded to a particular experimental condition. The opaque barriers also limited visual access to surrounding environmental cues, although we acknowledge that the broader physical environment could not be fully standardized across breeding facilities.

The arena floor was not routinely cleaned between individual trials or puppies unless a puppy urinated or defecated, in which case the affected area was cleaned with a detergent solution before testing continued.

Two identical stainless-steel bowls were used throughout the procedure, but only one bowl was present in the arena during any individual presentation. The bowl presented at the non-rewarded locations (N and A) was empty but was rubbed with the food reward before presentation, so that it carried food-related olfactory cues comparable to those of the rewarded condition without providing access to food. Between puppies, both bowls were thoroughly washed to remove residual olfactory cues. Before subsequent use, the bowl presented at non-rewarded locations was again rubbed with the food reward to restore standardized food-related olfactory cues without providing access to food.

The starting point and the three bowl locations were marked on the floor with adhesive tape. Three bowl locations were defined: positive (P), negative (N), and ambiguous (A); the latter was placed midway between P and N, 1.5 m from each. The P, N, and A labels indicated three alternative target positions and did not represent simultaneously presented bowls; only one bowl was present in the arena during each individual presentation. In this study, “cue location” refers exclusively to the spatial position of the bowl (P, N, or A); no additional visual cue was used. All three locations were 3 m from the starting point.

All sessions were video recorded with a GoPro camera mounted on a tripod positioned behind the starting point, so that the whole test area and the three locations were within the field of view.

**Figure 1:**
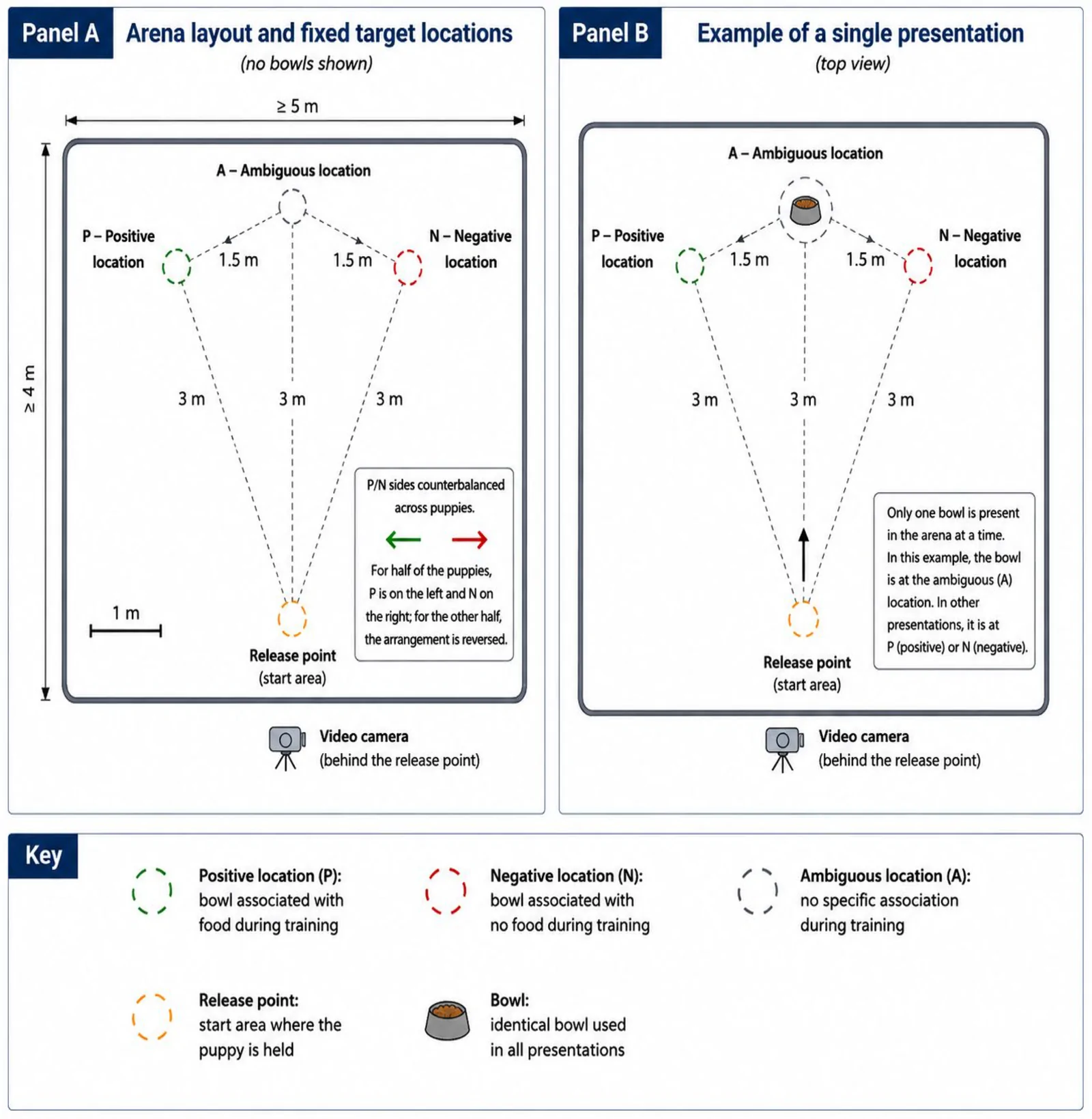
Schematic representation of the experimental setting and a single-bowl presentation. Panel (**A**) shows the arena dimensions and the three fixed target locations: positive (P), negative (N), and ambiguous (A). These markers represent alternative bowl locations; only one bowl was present in the arena during each presentation. All locations were 3 m from the release point, with A positioned midway between P and N, 1.5 m from each. The assignment of P and N to the left and right sides was counterbalanced across puppies. Panel (**B**) shows an example of a single presentation with the bowl positioned at A; in other presentations, the bowl was placed at P or N. Experimenter 1 held and released the puppy at the release point. The video camera was positioned behind the release point. The black arrow indicates the puppy’s direction of approach after release.

The spatial arrangement followed the judgment bias paradigm previously applied to dogs [16,23,28], in which the bowl locations are placed along an arc equidistant from the release point. Distances were reduced with respect to those studies (3 m instead of 4 m) and a single ambiguous location instead of three was used to account for the age and body size of the subjects and to keep the total duration of the procedure compatible with the attention span of 60-day-old puppies (Figure 1).

**Figure 2:**
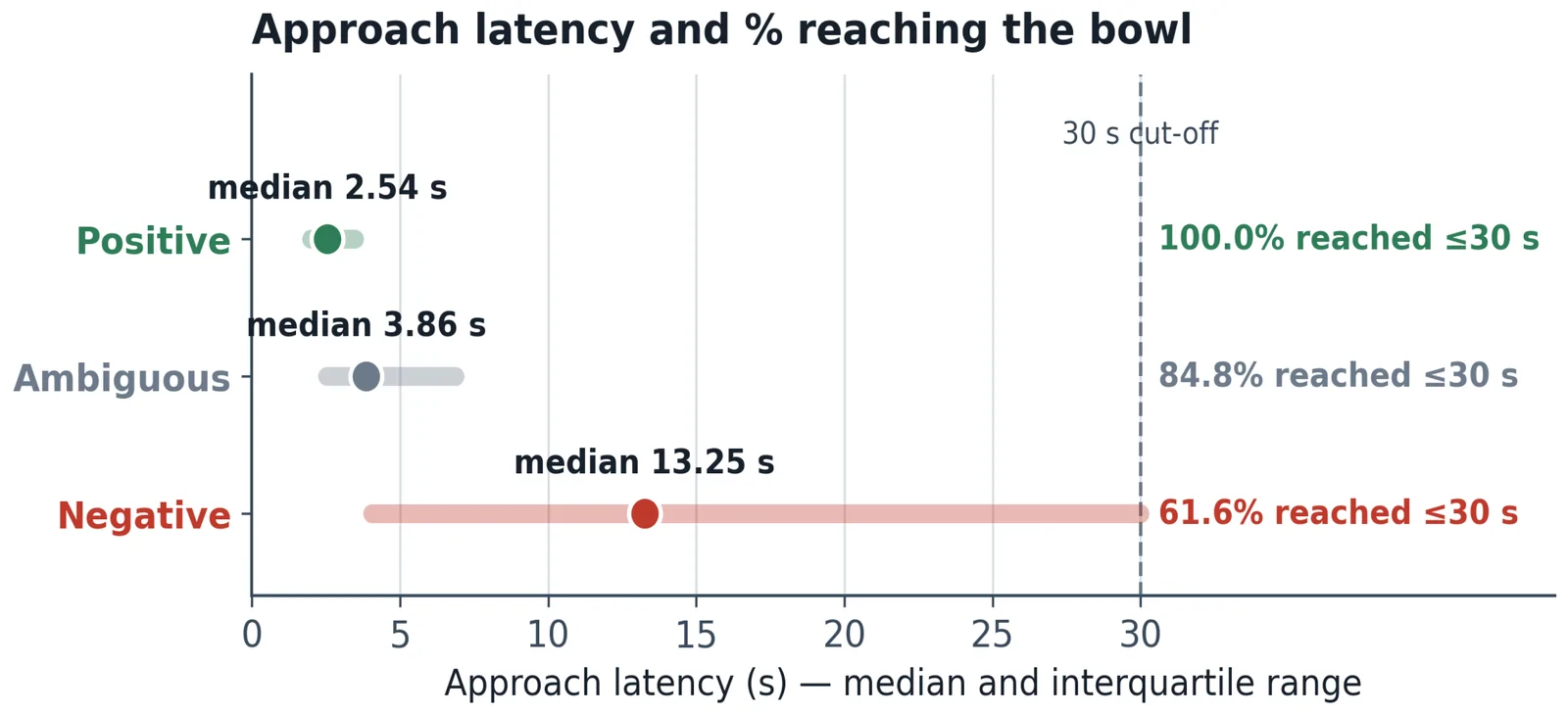
Approach responses in the spatial judgment-bias test (Trials 1 and 2; 476 observations from 80 puppies). Filled circles show the median approach latency and shaded bars the interquartile range for the positive, ambiguous and negative cue locations; the percentage of observations resulting in a successful approach within the 30-s cut-off is given at right. Medians and IQRs refer to observed or censoring times: for the negative cue the upper quartile coincides with the 30-s cut-off because 38.4% of observations were right-censored (non-approaches).

Generative AI tools were used as graphical aids in the preparation and refinement of Figure 1 and Figure 2. For Figure 1, all experimental elements, spatial relationships, dimensions, and labels were specified by the authors on the basis of the experimental protocol. For Figure 2, the graphical representation was based exclusively on the study data and statistical results provided by the authors. All graphical outputs were carefully reviewed and verified by the authors.

### 2.3. Habituation

Each puppy underwent a 10-min habituation phase in which it was allowed to explore the arena freely and to eat from the bowl, which was placed at the location subsequently designated as the P location. This placement was intended to facilitate familiarization with the feeding procedure and subsequent acquisition of the rewarded contingency. Because this procedure could potentially promote an early side association, the side assigned to the positive and negative locations was randomly counterbalanced across puppies to avoid a systematic spatial bias at the group level.

### 2.4. Release Procedure

The same standardized procedure was used in all training and test trials. Before each individual presentation, Experimenter 1 remained inside the enclosure and held the puppy on the ground, at the release point, facing away from the arena, without speaking or stroking the animal, while Experimenter 2 remained outside the pen and reached over the boundary of the enclosure to place the bowl at the required location (P, N, or A) for each individual presentation. Experimenter 2 then moved out of the puppy’s sight, behind a visual barrier, before the puppy was turned towards the arena and released, so that no person other than the handler was visible for the duration of the trial. This arrangement was adopted to minimize the potential influence of researcher presence on the puppies’ behavior, which has been identified as a source of bias in canine judgment bias protocols [23,24].

The puppy was subsequently turned towards the arena and briefly held at the release point to direct its attention towards the test area. The stopwatch started at the moment of release. Experimenter 1 was aware of the cue condition and was therefore not blinded to bowl location. To minimize unintentional signaling, Experimenter 1 followed the same standardized procedure in all trials, refraining from speaking to or stroking the puppy and providing no verbal or social cue at release. Once the puppy had touched the bowl, or once the maximum trial duration had elapsed, it was picked up by Experimenter 1 and returned to the waiting area; recall was never used and the puppy was never reinforced for returning, in order to avoid introducing an additional source of reinforcement.

### 2.5. Training

Training consisted of successive single-bowl presentations at P and N locations; only one bowl was present in the arena during each presentation. Training was conducted on the same day as habituation and testing, when puppies were 60 days old. No fixed inter-presentation interval was imposed; presentations were separated only by the time required to retrieve the puppy and reposition the bowl for the subsequent presentation. Training began with a fixed block of six rewarded presentations at the positive location, intended to establish contingency and to encourage participation, followed by two unrewarded presentations at the negative location. The order of positive and negative presentations following this opening block was determined by drawing lots, using the same procedure adopted for the testing order of the puppies, with a separate sequence drawn for each puppy; any sequence in which the same location occurred more than twice consecutively was discarded and redrawn.

Each presentation at the positive location was paired with the temporally adjacent presentation at the negative location, and a pair was scored as correct when the latency to the positive location was shorter than the latency to the negative location. Training was considered complete when three consecutive pairs were scored as correct, irrespective of the order in which the two locations were presented within each pair. Including the opening block, a maximum of 25 training presentations were allowed, and puppies not reaching the criterion within this limit were excluded from the test phase. All puppies included in the analyses reached the criterion, after a median of 15 presentations.

### 2.6. Judgment Bias Test

In the test phase, the ambiguous location was introduced midway between the positive and negative locations; it was never rewarded, and the contingencies at the positive and negative locations were unchanged.

Each test trial comprised three consecutive single-bowl presentations, one at each target location, in the standardized sequence P–N–A, beginning with the rewarded location to support continued task engagement. Only one bowl was present in the arena during each presentation. The same sequence was applied consistently to all puppies. Up to three trials were performed per puppy (P1, N1, A1; P2, N2, A2; P3, N3, A3). Trials 1 and 2 were completed by 100% and 97.5% of the puppies respectively, whereas the third trial was performed only when the puppy still appeared attentive and not fatigued; testing was discontinued otherwise.

### 2.7. Behavioral Measures

Latency to reach the target location was defined as the interval between the moment the puppy was released at the starting point and the moment it placed its head inside the bowl or touched the rim with its muzzle. Latencies were recorded in real time with a stopwatch by the same observer throughout the study and were subsequently verified by reviewing the video recordings; no additional behavioral variables were coded from the videos. The maximum trial duration was 30 s; puppies that did not reach the bowl within this interval were recorded as not having reached the target, and the corresponding observation was treated as right-censored at 30 s.

### 2.8. Statistical Analysis

Statistical analyses were performed using Jamovi version 2.6 [35] and R version 4.5.3 [36], with the survival package version 3.8-6 [37] and coxme package version 2.2-22 [38].

Latency data were summarized as medians and interquartile ranges because of their asymmetric distribution. For descriptive summaries only, observations right-censored at 30 s were represented by their censoring time. Accordingly, medians and interquartile ranges refer to observed or censoring times rather than to exact uncensored latency distributions. In the inferential analyses, puppies that did not reach the target location within 30 s were treated as right-censored observations rather than as having an exact latency of 30 s.

The primary analysis included Trials 1 and 2, which were available for 100% and 97.5% of the puppies respectively.

Approach latencies were analyzed using mixed-effects Cox proportional hazards models. Time to reach the target location was used as the outcome, with puppies that reached the location within the 30-s observation period coded as events and puppies that did not reach it coded as right-censored observations. The survival response was specified in R as Surv (latency, reached), with reached coded as 1 for successful approaches and 0 for censored observations.

Cue location (positive, ambiguous, or negative), trial number, and sex were included as fixed effects. Puppy identity and litter identity were included as random intercepts to account, respectively, for repeated observations from the same puppy and for the non-independence of puppies belonging to the same litter.

A cue-by-trial interaction was initially included to determine whether differences among cue locations varied between Trials 1 and 2. The model including the interaction was compared with the corresponding additive model using a likelihood-ratio test. As the interaction did not significantly improve model fit, the more parsimonious additive model was retained. Overall effects of cue location, trial number, and sex were evaluated by comparing the final model with corresponding reduced nested models.

Following the overall test of cue location, three planned pairwise comparisons were performed: positive versus ambiguous, positive versus negative, and ambiguous versus negative. The reference category of the cue factor was changed to obtain the relevant contrasts. To control the family-wise error rate, the three pairwise *p* values were adjusted using the sequential Holm procedure. Hazard ratios and their 95% confidence intervals were reported. In this context, a hazard ratio greater than 1 indicated a higher instantaneous rate of reaching the target location and, therefore, a faster approach. Statistical significance was set at *p* < 0.05, using Holm-adjusted *p* values for pairwise cue comparisons.

The proportional hazards assumption for the fixed effects of the final additive mixed-effects Cox model was assessed using scaled Schoenfeld residuals with the cox.zph() function applied directly to the fitted coxme model. Both term-specific and global tests were examined.

Model robustness was examined through sensitivity analyses restricted to the first trial and to puppies with complete data for all six observations collected during Trials 1 and 2. To assess the potential influence of site-level clustering, the primary mixed-effects Cox model was additionally refitted including breeding facility as a third random intercept, in addition to puppy and litter identity. Given the limited number of breeding facilities (*n* = 7) and the hierarchical nesting of litters within facilities, this model was considered an additional sensitivity analysis rather than part of the primary analysis.

Separately from the primary and sensitivity analyses, an exploratory analysis included all valid observations from Trial 3. This analysis was not part of the primary hypothesis testing and was used only to explore whether response patterns changed with additional repetition. Because Trial 3 was completed only by a non-random subset of puppies, this analysis was considered exploratory and its results were interpreted cautiously.

As a complementary analysis, the probability of reaching the target location within 30 s was analyzed using a binomial generalized linear mixed model with a logit link. This analysis was restricted to ambiguous and negative locations because all puppies reached a positive location, resulting in complete separation. Cue location, trial number, and sex were included as fixed effects, while puppy identity and litter identity were included as random intercepts.

The dataset used for the statistical analyses is provided as Supplementary Dataset S1.

## 3. Results

Of the 82 puppies initially enrolled, 80 provided valid test observations and constituted the final analytical sample, comprising 17 litters (Table 1).

The primary analysis included Trials 1 and 2 and comprised 476 valid individual presentations, defined as presentations for which latency and target-reaching status were successfully recorded: 240 from Trial 1 and 236 from Trial 2. Of these, 391 were successful approaches, in which the puppy reached the bowl within 30 s, whereas 85 were right-censored because the target was not reached within the 30-s observation period.

Approach latencies differed markedly among cue locations (Table 2A; Figure 2). Median latency was lowest for the positive location, intermediate for the ambiguous location, and highest for the negative location. Median latency was 2.54 s (IQR: 1.98–3.48) for the positive cue, 3.86 s (IQR: 2.51–6.86) for the ambiguous cue, and 13.25 s (IQR: 4.04–30.00) for the negative cue.

No timeouts occurred during single-bowl presentations at the positive (P) location. In contrast, when the single bowl was presented at the ambiguous (A) location, 24 of 158 presentations (15.2%) resulted in a timeout, whereas 61 of 159 presentations (38.4%) resulted in a timeout when the bowl was presented at the negative (N) location. These observations were right-censored at 30 s.

Responses to the positive cue were similar across repetitions, with median recorded times of 2.54 s (IQR: 1.97–3.23) in Trial 1 and 2.53 s (IQR: 2.00–3.51) in Trial 2. Median recorded times to the ambiguous cue were 3.90 s (IQR: 2.44–5.66) and 3.80 s (IQR: 2.57–17.20), respectively, indicating greater variability during Trial 2. For the negative cue, median latency increased from 10.70 s (IQR: 4.36–30.00) in Trial 1 to 15.00 s (IQR: 3.36–30.00) in Trial 2.

The initial model included the interaction between cue location and trial number. This interaction did not significantly improve model fit compared with the additive model (likelihood-ratio test: χ^2^ = 1.35, df = 2, *p* = 0.510) and was therefore removed.

In the final mixed-effects Cox model, cue location had a significant overall effect on approach latency (global likelihood-ratio test χ^2^ = 207.03, df = 2, *p* < 0.001). Compared with the positive location, puppies showed a lower approach rate towards both the ambiguous location (HR = 0.32, 95% CI: 0.25–0.41) and the negative location (HR = 0.11, 95% CI: 0.09–0.15). The approach rate towards the negative location was also lower than that towards the ambiguous location (HR = 0.36, 95% CI: 0.27–0.47). All three pairwise comparisons remained significant after Holm adjustment (Holm-adjusted *p* < 0.001, Table 2).

Trial number did not have a statistically significant overall effect (χ^2^ = 3.16, df = 1, *p* = 0.076). Nevertheless, responses tended to be slower during Trial 2 than during Trial 1 (HR = 0.83, 95% CI: 0.68–1.02). No significant effect of sex was observed (χ^2^ = 0.49, df = 1, *p* = 0.483; males vs. females: HR = 1.12, 95% CI: 0.82–1.52).

The estimated random-effect variance was 0.180 for puppy identity and 0.261 for litter identity, indicating additional heterogeneity both among individual puppies and among litters.

Tests based on scaled Schoenfeld residuals provided no evidence of violation of the proportional hazards assumption for cue location (*p* = 0.140), trial number (*p* = 0.160), sex (*p* = 0.340), or the model globally (*p* = 0.160).

Because all puppies reached the positive location within 30 s, the binomial mixed model was restricted to ambiguous and negative observations to avoid complete separation.

Cue location significantly affected the probability of reaching the target within 30 s (χ^2^ = 22.29, *p* < 0.001). The odds of reaching the negative location were lower than those of reaching the ambiguous location (OR = 0.24, 95% CI: 0.13–0.43). Neither trial number (χ^2^ = 0.73, *p* = 0.394) nor sex (χ^2^ = 0.72, *p* = 0.398) significantly affected approach probability.

When the analysis was restricted to Trial 1, cue location remained strongly associated with approach latency (χ^2^ = 116.31, df = 2, *p* < 0.001). Compared with the positive location, approach rates were lower for both the ambiguous cue (HR = 0.34, 95% CI: 0.24–0.48) and the negative cue (HR = 0.10, 95% CI: 0.07–0.16). The negative location was also approached more slowly than the ambiguous location (HR = 0.30, *p* < 0.001). Sex was not significant (*p* = 0.368).

The complete-case analysis, restricted to the 78 puppies with all six observations from Trials 1 and 2, produced estimates closely comparable with those obtained in the full analytical sample. The hazard ratios were 0.33 for the ambiguous versus positive comparison and 0.11 for the negative versus positive comparison. Trial number remained non-significant (*p* = 0.057), as did sex (*p* = 0.437).

In an additional sensitivity analysis, breeding facility was included as a third random intercept. The additional facility-level variance, after accounting for puppy and litter, was very small (variance = 0.0004; SD = 0.020), while the puppy- and litter-level variance estimates remained essentially unchanged (0.180 and 0.261, respectively). Fixed-effect estimates were also virtually identical to those of the primary model (A vs. P: HR = 0.319; N vs. P: HR = 0.114; Trial 2 vs. Trial 1: HR = 0.831; male vs. female: HR = 1.117), indicating that the main findings were robust to additional adjustment for breeding facility.

The exploratory analysis, including all available observations from Trials 1–3, comprised 593 observations from 80 puppies. Cue location remained strongly associated with approach latency, and the cue-by-trial interaction was not significant, indicating that the relative ordering of responses to positive, ambiguous, and negative cues was broadly consistent across trials. Trial number showed a significant overall effect (χ^2^ = 7.36, df = 2, *p* = 0.025). Responses during Trial 2 did not differ significantly from those during Trial 1 (HR = 0.83, *p* = 0.078), whereas responses during Trial 3 were slower than during Trial 1 (HR = 0.70, 95% CI: 0.53–0.92, *p* = 0.010). Because Trial 3 was completed only by a non-random subset of puppies, this analysis was considered exploratory, and its results were interpreted cautiously; it was not used to support the primary conclusions.

## 4. Discussion

The present study investigated judgment bias responses in domestic dog puppies at 60 days of age. Puppies showed a clear graded response across cue locations: the positive location was approached most rapidly, the ambiguous location elicited an intermediate response, and the negative location was approached most slowly and was associated with the highest proportion of non-approaches. This pattern remained robust when the analysis was restricted to the first trial and to puppies with complete observations. Neither sex nor repetition across the first two trials significantly affected performance.

The graded response across cue locations is consistent with the rationale of judgment bias tasks [4,5,6] and with findings reported in adult dogs using comparable spatial paradigms [16,23]. Similar monotonic response gradients have also been described in other species [39]. The short and consistent latencies to the positive location, together with the longer responses and frequent non-approaches to the negative location, indicate that the puppies discriminated between the learned contingencies. Their intermediate response to the ambiguous location suggests that they treated this cue as neither fully equivalent to the positive nor to the negative location, supporting the feasibility of assessing decision-making under ambiguity at approximately eight weeks of age.

However, the group-level response to the ambiguous cue should not be interpreted as evidence that individual puppies possessed stable optimistic or pessimistic traits. Judgment bias performance may reflect both transient affective states and more persistent individual characteristics [4,6]. In adult dogs, responses to ambiguous cues have been associated with behavioral and personality traits, including sociability, fearfulness, and separation-related behaviors [16,22], and comparable associations have been reported in other species [40]. Establishing judgment bias as an individual characteristic requires evidence of repeatability over time and across contexts [6,28]. Although temporal consistency has been reported in laboratory rodents [41,42], repeatability of cognitive performance is generally modest across taxa [43]. Longitudinal assessment is therefore needed to determine whether responses observed in puppies are stable across development and associated with other dimensions of temperament [34].

Responses were broadly stable across Trials 1 and 2, as indicated by the absence of a significant cue-by-trial interaction. In contrast, the exploratory analysis including Trial 3 suggested a general reduction in approach rate. This finding should be interpreted cautiously because Trial 3 was completed only by a non-random subset of puppies that remained sufficiently attentive and not excessively fatigued. Repeated exposure may also reduce cue ambiguity through learning, as previously reported in canine and other judgment bias studies [27,28]. Fatigue, habituation, and declining food motivation may additionally have contributed to slower responses. These considerations support the decision to base the primary analysis on the first two trials and to treat Trial 3 as exploratory.

No significant sex differences were detected in either approach latency or the probability of reaching the target. This is consistent with previous findings in two-month-old puppies assessed in a standardized open-field test [44]. Nevertheless, the absence of a significant effect should not be interpreted as evidence that sex cannot influence judgment bias, as sex has been identified as a moderator in a meta-analysis of judgment bias studies [7]. Moreover, the present study was not specifically designed or powered to detect small sex-related differences, and possible interactions with breed, development, experience, or later hormonal maturation cannot be excluded.

Variation was detected at both puppy and litter levels, with somewhat greater estimated heterogeneity among litters. Litter effects have previously been reported in behavioral assessments of young puppies [44,45]. Puppies within the same litter share genetic background, prenatal conditions, maternal care, early environment, and management, all of which may contribute to behavioral variation. Maternal care has been associated with puppy behavior at approximately 58–60 days of age and with later temperament [46,47,48], while litter size and maternal parity may also influence behavioral development [49]. Moreover, some breeding facilities contributed more than one litter, and several breeds were represented by only one or a small number of litters. Consequently, breed was partly confounded with litter membership, and breed, litter, maternal, management, and breeding-facility effects could not be fully disentangled in the present design.

Future studies specifically addressing breed- or body-size-related differences should include multiple independent litters within each breed and direct measures of body size.

A methodological strength of the study was the treatment of observations reaching the 30-s limit as right-censored rather than as exact latencies. This allowed non-approaches to be retained appropriately in the mixed-effects Cox model and was particularly relevant for the negative cue, for which censored observations were frequent. Survival analysis has previously been applied to approach latencies in canine judgment bias tasks for the same reason [18]. The complementary binomial analysis produced a consistent pattern, showing that puppies were also less likely to reach the negative than the ambiguous location within the observation period.

Several limitations should be acknowledged. First, because testing was conducted at different breeding facilities and under both indoor and outdoor conditions, broader environmental characteristics and ambient temperature could not be fully standardized. Second, the study did not include experimental manipulation intended to induce positive or negative affective states. Consequently, the results demonstrate differences in responses to cue locations but do not establish the construct validity of the test as a measure of affective valence in puppies [6,50]. Third, only one ambiguous location was examined, limiting the possibility of evaluating a complete generalization gradient between the positive and negative cues; designs employing multiple ambiguous locations have revealed symmetrical response gradients in other species [27,39]. Fourth, factors such as individual food motivation, general locomotor speed, exploration tendency, and recent experiences were not independently quantified and may have contributed to approach behavior. Judgment bias performance can reflect not only affective state but also learning, motivation and individual behavioral characteristics [4,23,26].

Fifth, Experimenter 1 was not blinded to cue condition. Although handling and release were standardized and no verbal or social cues were provided, subtle unintentional human signaling cannot be entirely excluded.

Finally, the use of the same P–N–A sequence for all puppies ensured consistency of the testing procedure. However, because the order of cue presentation was not varied, a possible influence of presentation sequence on approach responses cannot be entirely ruled out. Future studies could examine whether counterbalancing cue order affects the response pattern observed in young puppies.

An additional value of testing puppies at 60 days of age is that it provides a standardized behavioral baseline immediately before adoption. At this stage, puppies are still living with their dam and littermates and have not yet been exposed to the diverse household environments, management practices, training routines, and human–dog interactions that follow placement in a new home. Repeating the same or comparable assessments after adoption could therefore help distinguish relatively stable individual differences from changes associated with subsequent experience. This approach may be particularly informative for studying emotional development and welfare, as early-life factors such as maternal care and litter characteristics have already been associated with behavioral development and later temperament in dogs [46,47,48,49]. Combining pre-adoption judgment bias assessment with longitudinal post-adoption measures could therefore help clarify how early and later environmental experiences interact with individual characteristics in shaping responses to ambiguity, adaptation, and welfare.

Nevertheless, including breeding facility as an additional random intercept in a sensitivity analysis produced virtually unchanged fixed-effect estimates and negligible additional facility-level variance after accounting for puppy and litter, indicating that the main findings were robust to additional adjustment for site-level clustering.

Breed and body size may therefore have contributed to variation in approach latency; however, breed was not included as an additional fixed effect because the study was not designed to estimate breed-specific effects and several breeds were represented by only one or a small number of litters. Future studies should include larger numbers of independent litters within breeds, standardized repeated assessments, and independent measures of temperament, motivation, body size, and environmental conditions. Longitudinal re-testing would be particularly valuable for determining whether responses observed at 60 days are repeatable later in development and associated with subsequent behavioral characteristics or welfare outcomes [34]. Protocol variants designed to preserve cue ambiguity across repeated assessments, such as probability-based approaches, may be particularly useful in this context [51].

## 5. Conclusions

In conclusion, puppies at 60 days of age showed clear and graded behavioral responses to positive, ambiguous, and negative cue locations. The ambiguous cue elicited an intermediate response, while the negative cue was associated with substantially slower and less frequent approaches. These results support the feasibility of applying a spatial judgment bias task during early canine development. Although the present cross-sectional study does not allow responses of individual puppies to be interpreted as stable optimistic or pessimistic traits, their possible association with other dimensions of temperament warrants further investigation. Longitudinal re-testing of puppies after adoption could help determine whether individual differences in responses to ambiguity are stable across development or are influenced by the post-adoption dog–human environment. If such responses prove to be repeatable and associated with other temperament characteristics, their assessment during puppyhood may contribute to a more comprehensive understanding of individual temperament and, potentially, to strategies aimed at promoting adaptation and welfare after adoption.

## Figures and Tables

**Table 1:** Characteristics of the puppies included in the behavioral analyses (*n* = 80).

Characteristic	Value, *n* (%)	Litters, *n*
Overall sample	80 (100.0)	17
Age at testing, days	60	—
**Sex**		
Female	46 (57.5)	—
Male	34 (42.5)	—
**Breed**		
American Staffordshire Terrier	3 (3.8)	1
Toy Poodle	9 (11.3)	2
Dachshund	11 (13.8)	2
Border Collie	13 (16.3)	3
Boston Terrier	1 (1.3)	1
Cocker Spaniel	7 (8.8)	1
Golden Retriever	5 (6.3)	1
Belgian Malinois	13 (16.3)	3
Australian Shepherd	13 (16.3)	2
Segugio Maremmano	5 (6.3)	1
**Test completion**		
Trial 1: complete P–N–A set	80 (100.0)	—
Trial 2: complete P–N–A set	78 (97.5)	—
Trial 3: complete P–N–A set	39 (48.8)	—

P, positive location; N, negative location; A, ambiguous location.

**Table 2:** Descriptive approach responses by cue location and results of the primary mixed-effects Cox proportional hazards model.

(**A**) Descriptive results for Trials 1 and 2					
**Cue Location**	**Observations, *n***	**Reached Target, *n* (%)**	**Timeout, *n* (%)**	**Median Recorded Time, s**	**IQR, s**
Positive (P)	159	159 (100.0)	0 (0.0)	2.54	1.98–3.48
Negative (N)	159	98 (61.6)	61 (38.4)	13.25	4.04–30.00
Ambiguous (A)	158	134 (84.8)	24 (15.2)	3.86	2.51–6.86
(**B**) Fixed effects and planned cue contrasts					
**Predictor or Contrast**	**B**	**SE**	**Hazard ratio (95% CI)**		***p* Value**
Negative vs. positive	−2.171	0.153	0.114 (0.085–0.154)		<0.001
Ambiguous vs. positive	−1.142	0.130	0.319 (0.247–0.412)		<0.001
Negative vs. ambiguous	−1.029	0.143	0.357 (0.270–0.473)		<0.001
Trial 2 vs. Trial 1	−0.185	0.104	0.831 (0.677–1.019)		0.076
Male vs. female	0.111	0.158	1.118 (0.820–1.523)		0.483
(**C**) Random effects					
**Random Intercept**	**Variance**		**Standard Deviation**		
Puppy identity	0.180		0.424		
Litter identity	0.261		0.511		

Note. Hazard ratios below 1 indicate a lower instantaneous approach rate and therefore a slower approach relative to the stated reference category. *p* values for the three planned cue contrasts were adjusted using the Holm procedure; those for trial and sex were unadjusted. The lower number of ambiguous observations reflects one puppy for whom testing was discontinued after the P2 and N2 presentations, before A2. B, regression coefficient; IQR, interquartile range; CI, confidence interval; SE, standard error.

## Data Availability

The original contributions presented in this study are included in the article/Supplementary Material. Further inquiries can be directed to the corresponding author.

## Supplementary Materials

The following supporting information can be downloaded at: https://www.mdpi.com/article/10.3390/vetsci13080828/s1, Dataset S1: Raw dataset used for the statistical analyses.
