# Supplementary material for: Judgment Bias Responses in Domestic Dog Puppies at 60 Days of Age
Source: Vet Sci. 2026 Aug 19;13(8):828. doi: 10.3390/vetsci13080828 (PMC13517232; doi:10.3390/vetsci13080828)
Supplement: Supplementary file 1 [file vetsci-13-00828-s001.zip › vetsci-4491272-supplementary.pdf]

Puppy_ID	itter_I	Sex	Breed	Cue	Trial	atency_
P001	L003	M	American Staffordshire Terrier	P	1	2.51
P001	L003	M	American Staffordshire Terrier	P	2	2.06
P001	L003	M	American Staffordshire Terrier	P	3	
P001	L003	M	American Staffordshire Terrier	N	1	30.00
P001	L003	M	American Staffordshire Terrier	N	2	30.00
P001	L003	M	American Staffordshire Terrier	N	3	
P001	L003	M	American Staffordshire Terrier	A	1	4.21
P001	L003	M	American Staffordshire Terrier	A	2	3.36
P001	L003	M	American Staffordshire Terrier	A	3	
P002	L003	M	American Staffordshire Terrier	P	1	1.56
P002	L003	M	American Staffordshire Terrier	P	2	1.39
P002	L003	M	American Staffordshire Terrier	P	3	
P002	L003	M	American Staffordshire Terrier	N	1	30.00
P002	L003	M	American Staffordshire Terrier	N	2	30.00
P002	L003	M	American Staffordshire Terrier	N	3	
P002	L003	M	American Staffordshire Terrier	A	1	2.21
P002	L003	M	American Staffordshire Terrier	A	2	1.70
P002	L003	M	American Staffordshire Terrier	A	3	
P003	L003	F	American Staffordshire Terrier	P	1	5.19
P003	L003	F	American Staffordshire Terrier	P	2	2.88
P003	L003	F	American Staffordshire Terrier	P	3	
P003	L003	F	American Staffordshire Terrier	N	1	30.00
P003	L003	F	American Staffordshire Terrier	N	2	30.00
P003	L003	F	American Staffordshire Terrier	N	3	
P003	L003	F	American Staffordshire Terrier	A	1	2.46
P003	L003	F	American Staffordshire Terrier	A	2	2.68
P003	L003	F	American Staffordshire Terrier	A	3	
P004	L007	F	Australian Shepherd	P	1	7.58
P004	L007	F	Australian Shepherd	P	2	2.31
P004	L007	F	Australian Shepherd	P	3	3.18
P004	L007	F	Australian Shepherd	N	1	30.00
P004	L007	F	Australian Shepherd	N	2	8.90
P004	L007	F	Australian Shepherd	N	3	6.24
P004	L007	F	Australian Shepherd	A	1	5.10
P004	L007	F	Australian Shepherd	A	2	3.49
P004	L007	F	Australian Shepherd	A	3	30.00
P005	L007	F	Australian Shepherd	P	1	2.48
P005	L007	F	Australian Shepherd	P	2	4.31
P005	L007	F	Australian Shepherd	P	3	4.80
P005	L007	F	Australian Shepherd	N	1	30.00
P005	L007	F	Australian Shepherd	N	2	8.25
P005	L007	F	Australian Shepherd	N	3	30.00
P005	L007	F	Australian Shepherd	A	1	4.11
P005	L007	F	Australian Shepherd	A	2	4.12
P005	L007	F	Australian Shepherd	A	3	4.99
P006	L007	F	Australian Shepherd	P	1	2.87
P006	L007	F	Australian Shepherd	P	2	3.35
P006	L007	F	Australian Shepherd	P	3	4.03
P006	L007	F	Australian Shepherd	N	1	30.00
P006	L007	F	Australian Shepherd	N	2	30.00

P006	L007	F	Australian Shepherd	N	3	30.00
P006	L007	F	Australian Shepherd	A	1	4.11
P006	L007	F	Australian Shepherd	A	2	3.41
P006	L007	F	Australian Shepherd	A	3	30.00
P007	L007	F	Australian Shepherd	P	1	2.54
P007	L007	F	Australian Shepherd	P	2	1.75
P007	L007	F	Australian Shepherd	P	3	1.91
P007	L007	F	Australian Shepherd	N	1	10.30
P007	L007	F	Australian Shepherd	N	2	4.40
P007	L007	F	Australian Shepherd	N	3	30.00
P007	L007	F	Australian Shepherd	A	1	2.61
P007	L007	F	Australian Shepherd	A	2	14.48
P007	L007	F	Australian Shepherd	A	3	7.50
P008	L007	F	Australian Shepherd	P	1	3.97
P008	L007	F	Australian Shepherd	P	2	2.52
P008	L007	F	Australian Shepherd	P	3	3.51
P008	L007	F	Australian Shepherd	N	1	6.41
P008	L007	F	Australian Shepherd	N	2	30.00
P008	L007	F	Australian Shepherd	N	3	3.89
P008	L007	F	Australian Shepherd	A	1	4.55
P008	L007	F	Australian Shepherd	A	2	30.00
P008	L007	F	Australian Shepherd	A	3	4.37
P009	L007	F	Australian Shepherd	P	1	1.86
P009	L007	F	Australian Shepherd	P	2	2.21
P009	L007	F	Australian Shepherd	P	3	2.94
P009	L007	F	Australian Shepherd	N	1	30.00
P009	L007	F	Australian Shepherd	N	2	30.00
P009	L007	F	Australian Shepherd	N	3	30.00
P009	L007	F	Australian Shepherd	A	1	2.14
P009	L007	F	Australian Shepherd	A	2	2.44
P009	L007	F	Australian Shepherd	A	3	2.39
P010	L007	F	Australian Shepherd	P	1	2.23
P010	L007	F	Australian Shepherd	P	2	1.74
P010	L007	F	Australian Shepherd	P	3	2.04
P010	L007	F	Australian Shepherd	N	1	30.00
P010	L007	F	Australian Shepherd	N	2	30.00
P010	L007	F	Australian Shepherd	N	3	30.00
P010	L007	F	Australian Shepherd	A	1	3.28
P010	L007	F	Australian Shepherd	A	2	1.94
P010	L007	F	Australian Shepherd	A	3	3.11
P011	L010	F	Australian Shepherd	P	1	3.57
P011	L010	F	Australian Shepherd	P	2	2.78
P011	L010	F	Australian Shepherd	P	3	
P011	L010	F	Australian Shepherd	N	1	6.15
P011	L010	F	Australian Shepherd	N	2	30.00
P011	L010	F	Australian Shepherd	N	3	
P011	L010	F	Australian Shepherd	A	1	6.15
P011	L010	F	Australian Shepherd	A	2	18.45
P011	L010	F	Australian Shepherd	A	3	
P012	L010	F	Australian Shepherd	P	1	2.60
P012	L010	F	Australian Shepherd	P	2	2.87
P012	L010	F	Australian Shepherd	P	3	

P012	L010	F	Australian Shepherd	N	1	6.67
P012	L010	F	Australian Shepherd	N	2	15.55
P012	L010	F	Australian Shepherd	N	3	
P012	L010	F	Australian Shepherd	A	1	3.06
P012	L010	F	Australian Shepherd	A	2	2.01
P012	L010	F	Australian Shepherd	A	3	
P013	L010	F	Australian Shepherd	P	1	3.72
P013	L010	F	Australian Shepherd	P	2	4.70
P013	L010	F	Australian Shepherd	P	3	
P013	L010	F	Australian Shepherd	N	1	30.00
P013	L010	F	Australian Shepherd	N	2	30.00
P013	L010	F	Australian Shepherd	N	3	
P013	L010	F	Australian Shepherd	A	1	3.98
P013	L010	F	Australian Shepherd	A	2	5.83
P013	L010	F	Australian Shepherd	A	3	
P014	L010	F	Australian Shepherd	P	1	3.08
P014	L010	F	Australian Shepherd	P	2	3.53
P014	L010	F	Australian Shepherd	P	3	
P014	L010	F	Australian Shepherd	N	1	18.88
P014	L010	F	Australian Shepherd	N	2	18.04
P014	L010	F	Australian Shepherd	N	3	
P014	L010	F	Australian Shepherd	A	1	30.00
P014	L010	F	Australian Shepherd	A	2	2.59
P014	L010	F	Australian Shepherd	A	3	
P015	L010	F	Australian Shepherd	P	1	3.58
P015	L010	F	Australian Shepherd	P	2	2.88
P015	L010	F	Australian Shepherd	P	3	
P015	L010	F	Australian Shepherd	N	1	3.81
P015	L010	F	Australian Shepherd	N	2	11.02
P015	L010	F	Australian Shepherd	N	3	
P015	L010	F	Australian Shepherd	A	1	5.49
P015	L010	F	Australian Shepherd	A	2	3.73
P015	L010	F	Australian Shepherd	A	3	
P016	L010	F	Australian Shepherd	P	1	2.09
P016	L010	F	Australian Shepherd	P	2	2.44
P016	L010	F	Australian Shepherd	P	3	
P016	L010	F	Australian Shepherd	N	1	5.66
P016	L010	F	Australian Shepherd	N	2	30.00
P016	L010	F	Australian Shepherd	N	3	
P016	L010	F	Australian Shepherd	A	1	17.53
P016	L010	F	Australian Shepherd	A	2	3.49
P016	L010	F	Australian Shepherd	A	3	
P017	L006	F	Belgian Malinois	P	1	1.68
P017	L006	F	Belgian Malinois	P	2	2.27
P017	L006	F	Belgian Malinois	P	3	
P017	L006	F	Belgian Malinois	N	1	30.00
P017	L006	F	Belgian Malinois	N	2	3.11
P017	L006	F	Belgian Malinois	N	3	
P017	L006	F	Belgian Malinois	A	1	2.13
P017	L006	F	Belgian Malinois	A	2	30.00
P017	L006	F	Belgian Malinois	A	3	
P018	L006	M	Belgian Malinois	P	1	3.27

P018	L006	M	Belgian Malinois	P	2	5.04
P018	L006	M	Belgian Malinois	P	3	
P018	L006	M	Belgian Malinois	N	1	30.00
P018	L006	M	Belgian Malinois	N	2	30.00
P018	L006	M	Belgian Malinois	N	3	
P018	L006	M	Belgian Malinois	A	1	30.00
P018	L006	M	Belgian Malinois	A	2	30.00
P018	L006	M	Belgian Malinois	A	3	
P019	L006	F	Belgian Malinois	P	1	6.54
P019	L006	F	Belgian Malinois	P	2	1.62
P019	L006	F	Belgian Malinois	P	3	
P019	L006	F	Belgian Malinois	N	1	30.00
P019	L006	F	Belgian Malinois	N	2	6.97
P019	L006	F	Belgian Malinois	N	3	
P019	L006	F	Belgian Malinois	A	1	30.00
P019	L006	F	Belgian Malinois	A	2	30.00
P019	L006	F	Belgian Malinois	A	3	
P020	L012	F	Belgian Malinois	P	1	2.82
P020	L012	F	Belgian Malinois	P	2	1.47
P020	L012	F	Belgian Malinois	P	3	
P020	L012	F	Belgian Malinois	N	1	13.25
P020	L012	F	Belgian Malinois	N	2	30.00
P020	L012	F	Belgian Malinois	N	3	
P020	L012	F	Belgian Malinois	A	1	2.95
P020	L012	F	Belgian Malinois	A	2	18.09
P020	L012	F	Belgian Malinois	A	3	
P021	L012	F	Belgian Malinois	P	1	2.10
P021	L012	F	Belgian Malinois	P	2	2.30
P021	L012	F	Belgian Malinois	P	3	
P021	L012	F	Belgian Malinois	N	1	30.00
P021	L012	F	Belgian Malinois	N	2	30.00
P021	L012	F	Belgian Malinois	N	3	
P021	L012	F	Belgian Malinois	A	1	2.25
P021	L012	F	Belgian Malinois	A	2	1.40
P021	L012	F	Belgian Malinois	A	3	
P022	L012	M	Belgian Malinois	P	1	2.50
P022	L012	M	Belgian Malinois	P	2	9.66
P022	L012	M	Belgian Malinois	P	3	
P022	L012	M	Belgian Malinois	N	1	3.70
P022	L012	M	Belgian Malinois	N	2	1.65
P022	L012	M	Belgian Malinois	N	3	
P022	L012	M	Belgian Malinois	A	1	6.81
P022	L012	M	Belgian Malinois	A	2	2.43
P022	L012	M	Belgian Malinois	A	3	
P023	L012	M	Belgian Malinois	P	1	0.81
P023	L012	M	Belgian Malinois	P	2	1.70
P023	L012	M	Belgian Malinois	P	3	
P023	L012	M	Belgian Malinois	N	1	18.68
P023	L012	M	Belgian Malinois	N	2	22.00
P023	L012	M	Belgian Malinois	N	3	
P023	L012	M	Belgian Malinois	A	1	1.50
P023	L012	M	Belgian Malinois	A	2	2.46

P023	L012	M	Belgian Malinois	A	3	
P024	L013	F	Belgian Malinois	P	1	2.99
P024	L013	F	Belgian Malinois	P	2	2.28
P024	L013	F	Belgian Malinois	P	3	
P024	L013	F	Belgian Malinois	N	1	5.27
P024	L013	F	Belgian Malinois	N	2	4.38
P024	L013	F	Belgian Malinois	N	3	
P024	L013	F	Belgian Malinois	A	1	4.92
P024	L013	F	Belgian Malinois	A	2	3.08
P024	L013	F	Belgian Malinois	A	3	
P025	L013	F	Belgian Malinois	P	1	3.22
P025	L013	F	Belgian Malinois	P	2	2.10
P025	L013	F	Belgian Malinois	P	3	
P025	L013	F	Belgian Malinois	N	1	30.00
P025	L013	F	Belgian Malinois	N	2	30.00
P025	L013	F	Belgian Malinois	N	3	
P025	L013	F	Belgian Malinois	A	1	6.86
P025	L013	F	Belgian Malinois	A	2	30.00
P025	L013	F	Belgian Malinois	A	3	
P026	L013	F	Belgian Malinois	P	1	1.75
P026	L013	F	Belgian Malinois	P	2	1.35
P026	L013	F	Belgian Malinois	P	3	
P026	L013	F	Belgian Malinois	N	1	2.08
P026	L013	F	Belgian Malinois	N	2	1.51
P026	L013	F	Belgian Malinois	N	3	
P026	L013	F	Belgian Malinois	A	1	5.54
P026	L013	F	Belgian Malinois	A	2	2.22
P026	L013	F	Belgian Malinois	A	3	
P027	L013	M	Belgian Malinois	P	1	2.40
P027	L013	M	Belgian Malinois	P	2	2.97
P027	L013	M	Belgian Malinois	P	3	
P027	L013	M	Belgian Malinois	N	1	13.68
P027	L013	M	Belgian Malinois	N	2	2.65
P027	L013	M	Belgian Malinois	N	3	
P027	L013	M	Belgian Malinois	A	1	9.99
P027	L013	M	Belgian Malinois	A	2	3.58
P027	L013	M	Belgian Malinois	A	3	
P028	L013	F	Belgian Malinois	P	1	2.83
P028	L013	F	Belgian Malinois	P	2	16.53
P028	L013	F	Belgian Malinois	P	3	
P028	L013	F	Belgian Malinois	N	1	5.07
P028	L013	F	Belgian Malinois	N	2	7.00
P028	L013	F	Belgian Malinois	N	3	
P028	L013	F	Belgian Malinois	A	1	5.57
P028	L013	F	Belgian Malinois	A	2	4.92
P028	L013	F	Belgian Malinois	A	3	
P029	L013	M	Belgian Malinois	P	1	1.20
P029	L013	M	Belgian Malinois	P	2	2.61
P029	L013	M	Belgian Malinois	P	3	
P029	L013	M	Belgian Malinois	N	1	30.00
P029	L013	M	Belgian Malinois	N	2	3.45
P029	L013	M	Belgian Malinois	N	3	

P029	L013	M	Belgian Malinois	A	1	27.86
P029	L013	M	Belgian Malinois	A	2	23.08
P029	L013	M	Belgian Malinois	A	3	
P030	L002	M	Border Collie	P	1	1.59
P030	L002	M	Border Collie	P	2	2.66
P030	L002	M	Border Collie	P	3	2.59
P030	L002	M	Border Collie	N	1	2.28
P030	L002	M	Border Collie	N	2	3.08
P030	L002	M	Border Collie	N	3	2.76
P030	L002	M	Border Collie	A	1	2.53
P030	L002	M	Border Collie	A	2	1.56
P030	L002	M	Border Collie	A	3	2.34
P031	L002	F	Border Collie	P	1	3.74
P031	L002	F	Border Collie	P	2	4.74
P031	L002	F	Border Collie	P	3	4.48
P031	L002	F	Border Collie	N	1	14.43
P031	L002	F	Border Collie	N	2	30.00
P031	L002	F	Border Collie	N	3	16.26
P031	L002	F	Border Collie	A	1	4.72
P031	L002	F	Border Collie	A	2	5.85
P031	L002	F	Border Collie	A	3	10.97
P032	L002	F	Border Collie	P	1	3.06
P032	L002	F	Border Collie	P	2	3.04
P032	L002	F	Border Collie	P	3	5.32
P032	L002	F	Border Collie	N	1	5.21
P032	L002	F	Border Collie	N	2	30.00
P032	L002	F	Border Collie	N	3	6.12
P032	L002	F	Border Collie	A	1	2.19
P032	L002	F	Border Collie	A	2	3.99
P032	L002	F	Border Collie	A	3	3.52
P033	L002	F	Border Collie	P	1	2.63
P033	L002	F	Border Collie	P	2	2.49
P033	L002	F	Border Collie	P	3	4.44
P033	L002	F	Border Collie	N	1	4.64
P033	L002	F	Border Collie	N	2	2.94
P033	L002	F	Border Collie	N	3	7.55
P033	L002	F	Border Collie	A	1	2.94
P033	L002	F	Border Collie	A	2	2.08
P033	L002	F	Border Collie	A	3	3.06
P034	L002	M	Border Collie	P	1	2.06
P034	L002	M	Border Collie	P	2	2.53
P034	L002	M	Border Collie	P	3	2.98
P034	L002	M	Border Collie	N	1	6.07
P034	L002	M	Border Collie	N	2	2.16
P034	L002	M	Border Collie	N	3	2.75
P034	L002	M	Border Collie	A	1	3.96
P034	L002	M	Border Collie	A	2	3.29
P034	L002	M	Border Collie	A	3	3.66
P035	L004	F	Border Collie	P	1	2.54
P035	L004	F	Border Collie	P	2	3.78
P035	L004	F	Border Collie	P	3	
P035	L004	F	Border Collie	N	1	3.89

P035	L004	F	Border Collie	N	2	30.00
P035	L004	F	Border Collie	N	3	
P035	L004	F	Border Collie	A	1	3.58
P035	L004	F	Border Collie	A	2	3.88
P035	L004	F	Border Collie	A	3	
P036	L004	M	Border Collie	P	1	4.56
P036	L004	M	Border Collie	P	2	4.86
P036	L004	M	Border Collie	P	3	
P036	L004	M	Border Collie	N	1	7.74
P036	L004	M	Border Collie	N	2	30.00
P036	L004	M	Border Collie	N	3	
P036	L004	M	Border Collie	A	1	5.76
P036	L004	M	Border Collie	A	2	30.00
P036	L004	M	Border Collie	A	3	
P037	L004	M	Border Collie	P	1	2.94
P037	L004	M	Border Collie	P	2	2.79
P037	L004	M	Border Collie	P	3	
P037	L004	M	Border Collie	N	1	5.36
P037	L004	M	Border Collie	N	2	30.00
P037	L004	M	Border Collie	N	3	
P037	L004	M	Border Collie	A	1	2.24
P037	L004	M	Border Collie	A	2	4.79
P037	L004	M	Border Collie	A	3	
P038	L004	M	Border Collie	P	1	3.83
P038	L004	M	Border Collie	P	2	3.72
P038	L004	M	Border Collie	P	3	
P038	L004	M	Border Collie	N	1	4.33
P038	L004	M	Border Collie	N	2	9.56
P038	L004	M	Border Collie	N	3	
P038	L004	M	Border Collie	A	1	3.58
P038	L004	M	Border Collie	A	2	3.48
P038	L004	M	Border Collie	A	3	
P039	L015	F	Border Collie	P	1	2.16
P039	L015	F	Border Collie	P	2	3.18
P039	L015	F	Border Collie	P	3	2.41
P039	L015	F	Border Collie	N	1	2.75
P039	L015	F	Border Collie	N	2	6.41
P039	L015	F	Border Collie	N	3	12.40
P039	L015	F	Border Collie	A	1	4.03
P039	L015	F	Border Collie	A	2	2.56
P039	L015	F	Border Collie	A	3	3.13
P040	L015	M	Border Collie	P	1	1.88
P040	L015	M	Border Collie	P	2	2.38
P040	L015	M	Border Collie	P	3	2.69
P040	L015	M	Border Collie	N	1	30.00
P040	L015	M	Border Collie	N	2	14.81
P040	L015	M	Border Collie	N	3	7.44
P040	L015	M	Border Collie	A	1	1.78
P040	L015	M	Border Collie	A	2	2.59
P040	L015	M	Border Collie	A	3	3.10
P041	L015	M	Border Collie	P	1	4.79
P041	L015	M	Border Collie	P	2	4.04

P041	L015	M	Border Collie	P	3	3.94
P041	L015	M	Border Collie	N	1	14.97
P041	L015	M	Border Collie	N	2	11.93
P041	L015	M	Border Collie	N	3	16.60
P041	L015	M	Border Collie	A	1	5.09
P041	L015	M	Border Collie	A	2	6.85
P041	L015	M	Border Collie	A	3	5.97
P042	L015	F	Border Collie	P	1	3.69
P042	L015	F	Border Collie	P	2	6.44
P042	L015	F	Border Collie	P	3	4.28
P042	L015	F	Border Collie	N	1	4.37
P042	L015	F	Border Collie	N	2	13.60
P042	L015	F	Border Collie	N	3	17.00
P042	L015	F	Border Collie	A	1	3.84
P042	L015	F	Border Collie	A	2	3.60
P042	L015	F	Border Collie	A	3	3.78
P043	L005	F	Boston Terrier	P	1	2.56
P043	L005	F	Boston Terrier	P	2	5.66
P043	L005	F	Boston Terrier	P	3	
P043	L005	F	Boston Terrier	N	1	30.00
P043	L005	F	Boston Terrier	N	2	2.09
P043	L005	F	Boston Terrier	N	3	
P043	L005	F	Boston Terrier	A	1	2.99
P043	L005	F	Boston Terrier	A	2	30.00
P043	L005	F	Boston Terrier	A	3	
P044	L009	M	Cocker Spaniel	P	1	2.41
P044	L009	M	Cocker Spaniel	P	2	1.53
P044	L009	M	Cocker Spaniel	P	3	2.48
P044	L009	M	Cocker Spaniel	N	1	4.00
P044	L009	M	Cocker Spaniel	N	2	3.28
P044	L009	M	Cocker Spaniel	N	3	30.00
P044	L009	M	Cocker Spaniel	A	1	6.00
P044	L009	M	Cocker Spaniel	A	2	4.00
P044	L009	M	Cocker Spaniel	A	3	2.50
P045	L009	F	Cocker Spaniel	P	1	2.21
P045	L009	F	Cocker Spaniel	P	2	2.48
P045	L009	F	Cocker Spaniel	P	3	
P045	L009	F	Cocker Spaniel	N	1	4.03
P045	L009	F	Cocker Spaniel	N	2	30.00
P045	L009	F	Cocker Spaniel	N	3	
P045	L009	F	Cocker Spaniel	A	1	30.00
P045	L009	F	Cocker Spaniel	A	2	
P045	L009	F	Cocker Spaniel	A	3	
P046	L009	M	Cocker Spaniel	P	1	2.73
P046	L009	M	Cocker Spaniel	P	2	3.50
P046	L009	M	Cocker Spaniel	P	3	
P046	L009	M	Cocker Spaniel	N	1	4.15
P046	L009	M	Cocker Spaniel	N	2	4.45
P046	L009	M	Cocker Spaniel	N	3	
P046	L009	M	Cocker Spaniel	A	1	3.50
P046	L009	M	Cocker Spaniel	A	2	2.71
P046	L009	M	Cocker Spaniel	A	3	

P047	L009	M	Cocker Spaniel	P	1	3.75
P047	L009	M	Cocker Spaniel	P	2	3.53
P047	L009	M	Cocker Spaniel	P	3	
P047	L009	M	Cocker Spaniel	N	1	6.33
P047	L009	M	Cocker Spaniel	N	2	24.00
P047	L009	M	Cocker Spaniel	N	3	
P047	L009	M	Cocker Spaniel	A	1	4.60
P047	L009	M	Cocker Spaniel	A	2	30.00
P047	L009	M	Cocker Spaniel	A	3	
P048	L009	M	Cocker Spaniel	P	1	3.33
P048	L009	M	Cocker Spaniel	P	2	2.41
P048	L009	M	Cocker Spaniel	P	3	
P048	L009	M	Cocker Spaniel	N	1	30.00
P048	L009	M	Cocker Spaniel	N	2	30.00
P048	L009	M	Cocker Spaniel	N	3	
P048	L009	M	Cocker Spaniel	A	1	3.38
P048	L009	M	Cocker Spaniel	A	2	3.63
P048	L009	M	Cocker Spaniel	A	3	
P049	L009	F	Cocker Spaniel	P	1	2.78
P049	L009	F	Cocker Spaniel	P	2	3.77
P049	L009	F	Cocker Spaniel	P	3	
P049	L009	F	Cocker Spaniel	N	1	5.87
P049	L009	F	Cocker Spaniel	N	2	18.43
P049	L009	F	Cocker Spaniel	N	3	
P049	L009	F	Cocker Spaniel	A	1	3.13
P049	L009	F	Cocker Spaniel	A	2	3.92
P049	L009	F	Cocker Spaniel	A	3	
P050	L009	F	Cocker Spaniel	P	1	3.15
P050	L009	F	Cocker Spaniel	P	2	2.78
P050	L009	F	Cocker Spaniel	P	3	
P050	L009	F	Cocker Spaniel	N	1	30.00
P050	L009	F	Cocker Spaniel	N	2	30.00
P050	L009	F	Cocker Spaniel	N	3	
P050	L009	F	Cocker Spaniel	A	1	4.05
P050	L009	F	Cocker Spaniel	A	2	3.95
P050	L009	F	Cocker Spaniel	A	3	
P051	L017	F	Dachshund	P	1	8.50
P051	L017	F	Dachshund	P	2	5.68
P051	L017	F	Dachshund	P	3	6.07
P051	L017	F	Dachshund	N	1	30.00
P051	L017	F	Dachshund	N	2	30.00
P051	L017	F	Dachshund	N	3	30.00
P051	L017	F	Dachshund	A	1	5.63
P051	L017	F	Dachshund	A	2	30.00
P051	L017	F	Dachshund	A	3	7.31
P052	L017	F	Dachshund	P	1	2.57
P052	L017	F	Dachshund	P	2	2.13
P052	L017	F	Dachshund	P	3	2.31
P052	L017	F	Dachshund	N	1	30.00
P052	L017	F	Dachshund	N	2	2.56
P052	L017	F	Dachshund	N	3	2.56
P052	L017	F	Dachshund	A	1	3.22

P052	L017	F	Dachshund	A	2	2.28
P052	L017	F	Dachshund	A	3	3.15
P053	L017	F	Dachshund	P	1	2.69
P053	L017	F	Dachshund	P	2	3.46
P053	L017	F	Dachshund	P	3	5.03
P053	L017	F	Dachshund	N	1	10.37
P053	L017	F	Dachshund	N	2	30.00
P053	L017	F	Dachshund	N	3	30.00
P053	L017	F	Dachshund	A	1	4.62
P053	L017	F	Dachshund	A	2	30.00
P053	L017	F	Dachshund	A	3	13.25
P054	L017	M	Dachshund	P	1	1.94
P054	L017	M	Dachshund	P	2	3.50
P054	L017	M	Dachshund	P	3	2.50
P054	L017	M	Dachshund	N	1	16.03
P054	L017	M	Dachshund	N	2	30.00
P054	L017	M	Dachshund	N	3	6.78
P054	L017	M	Dachshund	A	1	30.00
P054	L017	M	Dachshund	A	2	3.12
P054	L017	M	Dachshund	A	3	30.00
P055	L017	M	Dachshund	P	1	5.97
P055	L017	M	Dachshund	P	2	
P055	L017	M	Dachshund	P	3	
P055	L017	M	Dachshund	N	1	5.33
P055	L017	M	Dachshund	N	2	
P055	L017	M	Dachshund	N	3	
P055	L017	M	Dachshund	A	1	30.00
P055	L017	M	Dachshund	A	2	
P055	L017	M	Dachshund	A	3	
P056	L018	M	Dachshund	P	1	2.32
P056	L018	M	Dachshund	P	2	2.81
P056	L018	M	Dachshund	P	3	2.31
P056	L018	M	Dachshund	N	1	30.00
P056	L018	M	Dachshund	N	2	2.81
P056	L018	M	Dachshund	N	3	30.00
P056	L018	M	Dachshund	A	1	2.90
P056	L018	M	Dachshund	A	2	30.00
P056	L018	M	Dachshund	A	3	30.00
P057	L018	F	Dachshund	P	1	2.25
P057	L018	F	Dachshund	P	2	2.56
P057	L018	F	Dachshund	P	3	2.22
P057	L018	F	Dachshund	N	1	30.00
P057	L018	F	Dachshund	N	2	30.00
P057	L018	F	Dachshund	N	3	30.00
P057	L018	F	Dachshund	A	1	3.57
P057	L018	F	Dachshund	A	2	2.60
P057	L018	F	Dachshund	A	3	3.13
P058	L018	F	Dachshund	P	1	3.10
P058	L018	F	Dachshund	P	2	1.79
P058	L018	F	Dachshund	P	3	2.15
P058	L018	F	Dachshund	N	1	1.22
P058	L018	F	Dachshund	N	2	2.47

P058	L018	F	Dachshund	N	3	30.00
P058	L018	F	Dachshund	A	1	2.66
P058	L018	F	Dachshund	A	2	30.00
P058	L018	F	Dachshund	A	3	8.87
P059	L018	F	Dachshund	P	1	2.28
P059	L018	F	Dachshund	P	2	2.00
P059	L018	F	Dachshund	P	3	2.10
P059	L018	F	Dachshund	N	1	3.40
P059	L018	F	Dachshund	N	2	30.00
P059	L018	F	Dachshund	N	3	30.00
P059	L018	F	Dachshund	A	1	2.40
P059	L018	F	Dachshund	A	2	4.25
P059	L018	F	Dachshund	A	3	2.09
P060	L018	F	Dachshund	P	1	1.97
P060	L018	F	Dachshund	P	2	2.62
P060	L018	F	Dachshund	P	3	2.28
P060	L018	F	Dachshund	N	1	17.75
P060	L018	F	Dachshund	N	2	14.97
P060	L018	F	Dachshund	N	3	30.00
P060	L018	F	Dachshund	A	1	25.53
P060	L018	F	Dachshund	A	2	8.56
P060	L018	F	Dachshund	A	3	11.78
P061	L018	M	Dachshund	P	1	1.63
P061	L018	M	Dachshund	P	2	1.53
P061	L018	M	Dachshund	P	3	1.81
P061	L018	M	Dachshund	N	1	2.10
P061	L018	M	Dachshund	N	2	2.72
P061	L018	M	Dachshund	N	3	15.47
P061	L018	M	Dachshund	A	1	1.72
P061	L018	M	Dachshund	A	2	19.72
P061	L018	M	Dachshund	A	3	3.13
P062	L016	M	Golden Retriever	P	1	2.28
P062	L016	M	Golden Retriever	P	2	2.00
P062	L016	M	Golden Retriever	P	3	2.85
P062	L016	M	Golden Retriever	N	1	5.38
P062	L016	M	Golden Retriever	N	2	4.06
P062	L016	M	Golden Retriever	N	3	30.00
P062	L016	M	Golden Retriever	A	1	2.03
P062	L016	M	Golden Retriever	A	2	3.69
P062	L016	M	Golden Retriever	A	3	2.94
P063	L016	M	Golden Retriever	P	1	2.59
P063	L016	M	Golden Retriever	P	2	3.81
P063	L016	M	Golden Retriever	P	3	3.56
P063	L016	M	Golden Retriever	N	1	2.72
P063	L016	M	Golden Retriever	N	2	18.19
P063	L016	M	Golden Retriever	N	3	6.37
P063	L016	M	Golden Retriever	A	1	5.28
P063	L016	M	Golden Retriever	A	2	4.56
P063	L016	M	Golden Retriever	A	3	25.88
P064	L016	M	Golden Retriever	P	1	2.35
P064	L016	M	Golden Retriever	P	2	2.38
P064	L016	M	Golden Retriever	P	3	5.59

P064	L016	M	Golden Retriever	N	1	3.25
P064	L016	M	Golden Retriever	N	2	3.28
P064	L016	M	Golden Retriever	N	3	3.31
P064	L016	M	Golden Retriever	A	1	2.16
P064	L016	M	Golden Retriever	A	2	2.50
P064	L016	M	Golden Retriever	A	3	3.22
P065	L016	F	Golden Retriever	P	1	1.97
P065	L016	F	Golden Retriever	P	2	2.18
P065	L016	F	Golden Retriever	P	3	
P065	L016	F	Golden Retriever	N	1	30.00
P065	L016	F	Golden Retriever	N	2	2.59
P065	L016	F	Golden Retriever	N	3	
P065	L016	F	Golden Retriever	A	1	2.78
P065	L016	F	Golden Retriever	A	2	4.00
P065	L016	F	Golden Retriever	A	3	
P066	L016	M	Golden Retriever	P	1	1.69
P066	L016	M	Golden Retriever	P	2	1.53
P066	L016	M	Golden Retriever	P	3	
P066	L016	M	Golden Retriever	N	1	11.81
P066	L016	M	Golden Retriever	N	2	2.90
P066	L016	M	Golden Retriever	N	3	
P066	L016	M	Golden Retriever	A	1	2.06
P066	L016	M	Golden Retriever	A	2	1.75
P066	L016	M	Golden Retriever	A	3	
P067	L011	M	Segugio Maremmano	P	1	1.55
P067	L011	M	Segugio Maremmano	P	2	2.02
P067	L011	M	Segugio Maremmano	P	3	
P067	L011	M	Segugio Maremmano	N	1	30.00
P067	L011	M	Segugio Maremmano	N	2	2.25
P067	L011	M	Segugio Maremmano	N	3	
P067	L011	M	Segugio Maremmano	A	1	2.28
P067	L011	M	Segugio Maremmano	A	2	30.00
P067	L011	M	Segugio Maremmano	A	3	
P068	L011	M	Segugio Maremmano	P	1	1.40
P068	L011	M	Segugio Maremmano	P	2	1.80
P068	L011	M	Segugio Maremmano	P	3	
P068	L011	M	Segugio Maremmano	N	1	1.25
P068	L011	M	Segugio Maremmano	N	2	6.10
P068	L011	M	Segugio Maremmano	N	3	
P068	L011	M	Segugio Maremmano	A	1	1.58
P068	L011	M	Segugio Maremmano	A	2	1.79
P068	L011	M	Segugio Maremmano	A	3	
P069	L011	F	Segugio Maremmano	P	1	0.95
P069	L011	F	Segugio Maremmano	P	2	1.07
P069	L011	F	Segugio Maremmano	P	3	
P069	L011	F	Segugio Maremmano	N	1	30.00
P069	L011	F	Segugio Maremmano	N	2	24.38
P069	L011	F	Segugio Maremmano	N	3	
P069	L011	F	Segugio Maremmano	A	1	1.86
P069	L011	F	Segugio Maremmano	A	2	1.14
P069	L011	F	Segugio Maremmano	A	3	
P070	L011	M	Segugio Maremmano	P	1	1.06

P070	L011	M	Segugio Maremmano	P	2	1.17
P070	L011	M	Segugio Maremmano	P	3	
P070	L011	M	Segugio Maremmano	N	1	10.99
P070	L011	M	Segugio Maremmano	N	2	1.65
P070	L011	M	Segugio Maremmano	N	3	
P070	L011	M	Segugio Maremmano	A	1	1.38
P070	L011	M	Segugio Maremmano	A	2	5.12
P070	L011	M	Segugio Maremmano	A	3	
P071	L011	F	Segugio Maremmano	P	1	1.30
P071	L011	F	Segugio Maremmano	P	2	1.28
P071	L011	F	Segugio Maremmano	P	3	
P071	L011	F	Segugio Maremmano	N	1	8.08
P071	L011	F	Segugio Maremmano	N	2	5.90
P071	L011	F	Segugio Maremmano	N	3	
P071	L011	F	Segugio Maremmano	A	1	1.58
P071	L011	F	Segugio Maremmano	A	2	1.75
P071	L011	F	Segugio Maremmano	A	3	
P072	L001	F	Toy Poodle	P	1	1.68
P072	L001	F	Toy Poodle	P	2	1.58
P072	L001	F	Toy Poodle	P	3	1.59
P072	L001	F	Toy Poodle	N	1	2.80
P072	L001	F	Toy Poodle	N	2	3.44
P072	L001	F	Toy Poodle	N	3	30.00
P072	L001	F	Toy Poodle	A	1	1.06
P072	L001	F	Toy Poodle	A	2	1.81
P072	L001	F	Toy Poodle	A	3	30.00
P073	L001	F	Toy Poodle	P	1	0.59
P073	L001	F	Toy Poodle	P	2	0.81
P073	L001	F	Toy Poodle	P	3	1.33
P073	L001	F	Toy Poodle	N	1	30.00
P073	L001	F	Toy Poodle	N	2	2.93
P073	L001	F	Toy Poodle	N	3	1.93
P073	L001	F	Toy Poodle	A	1	4.36
P073	L001	F	Toy Poodle	A	2	22.87
P073	L001	F	Toy Poodle	A	3	0.86
P074	L001	M	Toy Poodle	P	1	1.84
P074	L001	M	Toy Poodle	P	2	0.99
P074	L001	M	Toy Poodle	P	3	1.29
P074	L001	M	Toy Poodle	N	1	3.59
P074	L001	M	Toy Poodle	N	2	30.00
P074	L001	M	Toy Poodle	N	3	30.00
P074	L001	M	Toy Poodle	A	1	1.29
P074	L001	M	Toy Poodle	A	2	1.64
P074	L001	M	Toy Poodle	A	3	0.98
P075	L014	M	Toy Poodle	P	1	2.60
P075	L014	M	Toy Poodle	P	2	2.78
P075	L014	M	Toy Poodle	P	3	3.69
P075	L014	M	Toy Poodle	N	1	30.00
P075	L014	M	Toy Poodle	N	2	30.00
P075	L014	M	Toy Poodle	N	3	30.00
P075	L014	M	Toy Poodle	A	1	30.00
P075	L014	M	Toy Poodle	A	2	30.00

P075	L014	M	Toy Poodle	A	3	30.00
P076	L014	M	Toy Poodle	P	1	3.75
P076	L014	M	Toy Poodle	P	2	9.53
P076	L014	M	Toy Poodle	P	3	3.25
P076	L014	M	Toy Poodle	N	1	2.25
P076	L014	M	Toy Poodle	N	2	24.71
P076	L014	M	Toy Poodle	N	3	3.93
P076	L014	M	Toy Poodle	A	1	30.00
P076	L014	M	Toy Poodle	A	2	29.25
P076	L014	M	Toy Poodle	A	3	2.53
P077	L014	F	Toy Poodle	P	1	2.10
P077	L014	F	Toy Poodle	P	2	1.66
P077	L014	F	Toy Poodle	P	3	2.18
P077	L014	F	Toy Poodle	N	1	4.72
P077	L014	F	Toy Poodle	N	2	30.00
P077	L014	F	Toy Poodle	N	3	3.84
P077	L014	F	Toy Poodle	A	1	2.94
P077	L014	F	Toy Poodle	A	2	2.06
P077	L014	F	Toy Poodle	A	3	2.00
P078	L014	F	Toy Poodle	P	1	8.44
P078	L014	F	Toy Poodle	P	2	21.47
P078	L014	F	Toy Poodle	P	3	6.22
P078	L014	F	Toy Poodle	N	1	30.00
P078	L014	F	Toy Poodle	N	2	4.56
P078	L014	F	Toy Poodle	N	3	30.00
P078	L014	F	Toy Poodle	A	1	30.00
P078	L014	F	Toy Poodle	A	2	11.34
P078	L014	F	Toy Poodle	A	3	4.68
P079	L014	M	Toy Poodle	P	1	3.53
P079	L014	M	Toy Poodle	P	2	4.75
P079	L014	M	Toy Poodle	P	3	5.00
P079	L014	M	Toy Poodle	N	1	30.00
P079	L014	M	Toy Poodle	N	2	30.00
P079	L014	M	Toy Poodle	N	3	3.03
P079	L014	M	Toy Poodle	A	1	28.54
P079	L014	M	Toy Poodle	A	2	2.90
P079	L014	M	Toy Poodle	A	3	3.28
P080	L014	M	Toy Poodle	P	1	2.44
P080	L014	M	Toy Poodle	P	2	2.25
P080	L014	M	Toy Poodle	P	3	1.75
P080	L014	M	Toy Poodle	N	1	2.94
P080	L014	M	Toy Poodle	N	2	1.72
P080	L014	M	Toy Poodle	N	3	30.00
P080	L014	M	Toy Poodle	A	1	30.00
P080	L014	M	Toy Poodle	A	2	13.28
P080	L014	M	Toy Poodle	A	3	5.81
